# A91 OUTCOMES OF PATIENTS WITH PRIOR BIOLOGIC INTOLERANCE ARE BETTER THAN THOSE WITH BIOLOGIC FAILURE IN CLINICAL TRIALS OF INFLAMMATORY BOWEL DISEASE

**DOI:** 10.1093/jcag/gwae059.091

**Published:** 2025-02-10

**Authors:** S Samnani, E Wong, H Hamam, P Dulai, J Marshall, V Jairath, W Reinisch, N Narula

**Affiliations:** McMaster University, Hamilton, ON, Canada; McMaster University, Hamilton, ON, Canada; McMaster University, Hamilton, ON, Canada; Division of Gastroenterology, Northwestern University, Chicago, IL; McMaster University, Hamilton, ON, Canada; Department of Medicine, Division of Gastroenterology, Western University, London, ON, Canada; Department of Internal Medicine III, Division of Gastroenterology and Hepatology, Medical University of Vienna, Währinger Gürtel 18-20, Vienna, Austria; McMaster University, Hamilton, ON, Canada

## Abstract

**Background:**

Inflammatory bowel disease (IBD) trials often stratify patients by prior biologic exposure, including prior biologic failure or intolerance.

**Aims:**

This study aimed to assess clinical outcomes in IBD patients with prior biologic failure versus intolerance treated with ustekinumab or vedolizumab.

**Methods:**

A post-hoc analysis of ulcerative colitis (UC) and Crohn’s disease (CD) clinical trials for ustekinumab (UNITI, UNIFI) and vedolizumab (GEMINI-1, GEMINI-2) was performed. Clinical response, clinical remission, and endoscopic improvement (for UC) were compared among biologic naïve, biologic-failure, and biologic intolerant patients. Statistical analyses, including chi-square tests and logistic regression, were performed.

**Results:**

1178 UC and 1439 CD patients received either ustekinumab or vedolizumab. In UC, biologic intolerant patients exhibited higher clinical response (54.7% vs. 38.8%, aOR 1.87 [95% CI 0.93-3.73]), clinical remission (25.0% vs. 11.0%, aOR 2.84 [95% CI 1.47-5.49]), and endoscopic improvement (40.6% vs. 24.8%, aOR 2.76 [95% CI 1.28-5.94]) compared to biologic failure, with outcomes similar to biologic naïve patients. In biologic-intolerant CD patients, clinical response was similar between prior biologic failure and intolerance (34.2% vs 32.8%), but after adjustment for potential confounders, biologic intolerance was associated with higher odds of clinical response (aOR: 1.67, 95% CI 1.09-2.55), with no significant difference observed for clinical remission (aOR: 1.48, 95% CI 0.88-2.49).

**Conclusions:**

Improved treatment outcomes were generally observed in patients with biologic intolerance compared to failure, especially in UC, where outcomes were similar to biologic naïve patients. Future clinical trials should meticulously differentiate prior biologic failure versus intolerance to mitigate potential bias.

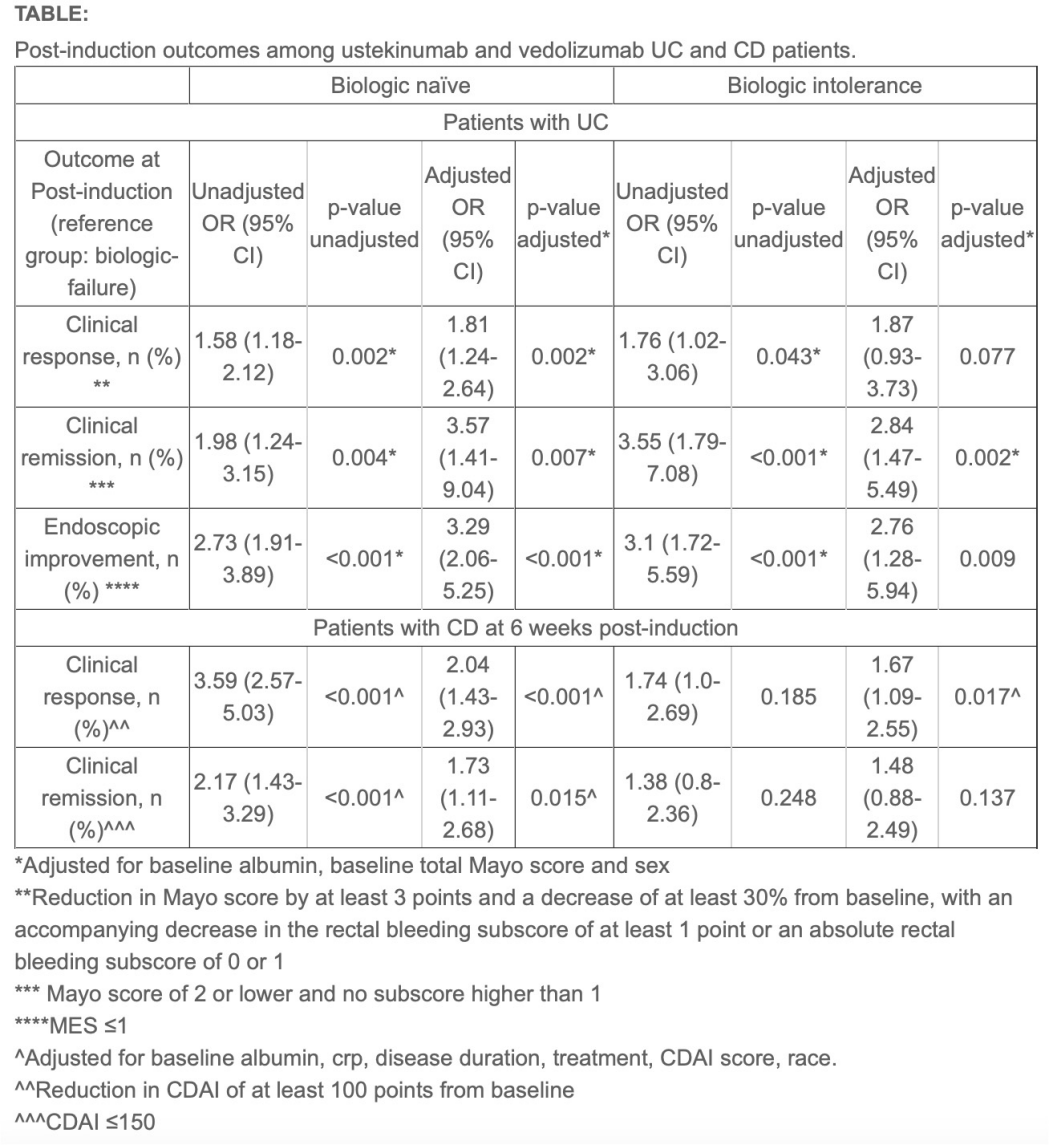

**Funding Agencies:**

None

